# Supplementary material for: Aberrant Methylation of Somatostatin Receptor 2 Gene Is Initiated in Aged Gastric Mucosa Infected with Helicobacter pylori and Consequential Gene Silencing Is Associated with Establishment of Inflammatory Microenvironment In Vitro Study
Source: Cancers (Basel). 2022 Dec 14;14(24):6183. doi: 10.3390/cancers14246183 (PMC9777158; doi:10.3390/cancers14246183)
Supplement: Supplementary file 1 [file cancers-14-06183-s001.zip › cancers-2018614-supplementary-final/cancers-2018614-supplementary.pdf]

Supplementary Information

Aberrant methylation of somatostatin receptor 2 gene is initiated in aged gastric mucosa infected with *Helicobacter pylori* and consequential gene silencing is associated with establishment of inflammatory microenvironment in vitro study

Hee-Jin Kim[1,†], Jong-Lyul Park[1,†], Byoung-Ha Yoon[2], Keeok Haam[1], Haejeong Heo[1,3], Jong Hwan Kim[2], Seon-Young Kim[2,3], Mirang Kim[1,3], Woo-Ho Kim[4], Sang-Il Lee[5], Kyu-Sang Song[6], Kwang-Dung Ahn[7], Yong Sung Kim[1,7]*

Table of content

1. Supplementary Tables

Supplementary Tables

Table S1. Primer sequences used in this study

Primer	Sequence(s)	T_m (℃)	Cycles	Product (bp)
(a) For RT-PCR and qRT-PCR for human SST and SSTR2 genes				
SST	F: 5′-CTAGAGTTTGACCAGCCAC-3′<br>R: 5′-GACAGATCTTCAGGTTCCAG-3′	60	35	268
SSTR2	F: 5′-CCCCTCACCATCATCTGTCT-3′<br>R: 5′-AGGTGAGGACCACCACAAAG-3′	62	33	247
ACTB	F: 5′-CAAGAGATGGCCACGGCTGCT-3'<br>R: 5′-TCCTTCTGCATCCTGTCGGCA-3'	60	25	191
(b) For bisulfite sequencing				
SST	F: 5′-AAAAGGGTTGGTGAGATTTGG-3'<br>R: 5′-AACACAACCCAAAACCAAAA-3'	56	40	252
SSTR2	F: 5′-GGGTTGGTTGGGTTAGTTTTAG-3′<br>R: 5′-CAAATACACACAAATACCCAAATAC-3′	60	35	393
(c) For pyrosequencing				
PCR for SST (19 CpGs)	F: 5′-Biotin-GAGGGAGAAGGTTGAGAGTATATAAGT-3′<br>R: 5′-ACACAACCCAAAACCAAAAC-3′	58	40	189
Sequencing primer	S: 5′-AAACCCAACTAAAAAATAACTAATC-3′			
PCR for SSTR2 (33 CpGs)	F: 5′-GGGGAGTGGGGAAATGTGT-3′<br>R: 5′-Biotin-CCAAACCTCAAACTAAAACTCT-3′	58	40	119
Sequencing primer	S: 5′-GGTTGGTTGGGTTAG-3′			
(d) For ChIP-PCR				
SST region 1	F: 5′-TGTGGGAGTGAAATTGTGGA-3'<br>R: 5′-GAAGACAGGGGAGGTGACAA-3'	60	35	160
region 2	F: 5′-ACTCTCCAGCTCGGCTTTC-3'<br>R: 5′-CTGCAGAAACTGACGGAGTC-3'	60	35	138
region 3	F: 5′-TCGGTCACAGTTCAGGTGAG-3'<br>R: 5′-GCTTCAGAGGTCTGCGTTTC-3'	60	35	150
SSTR2 region 1	F: 5′-GCTGACTGACGTGGCTACAG-3'<br>R: 5′-ATGAGCGCAGGGAGTGTAAA-3'	62	35	164
region 2	F: 5′-GAAGCCGCTGTGACGTAGC-3'<br>R: 5′-CTAGCCCCTCGCACGTAGA-3'	62	35	192
region 3	F: 5′-AGACCGGAGCTAGCGGATT-3'<br>R: 5′-GACCTAAGCTCAGGCACTCG-3'	62	35	178
(e) For gene cloning				
NotI-SSTR2-F<br>*NotI-SSTR2*-R	F: 5′-ATATTCTAGAGCCACCATGGACATGGCGGATGAGCCACTC-3′<br>R: 5′-ATATGCGGCCGCTCAGATACTGGTTTGGAGGTCTCC-3′	58	25	1138
(f) sgRNAs for human SSTR2				
Target 1	5′-CACCGATGGCGGATGAGCCACTCAA-3′<br>5′-AAACTTGAGTGGCTCATCCGCCATC-3′			
Target 2	5′-CACCGCACTCAATGGAAGCCACACA-3′<br>5′-AAACTGTGTGGCTTCCATTGAGTGC-3′			
Target 3	5′-CACCGTCCATTCCATTTGACCTCAA-3′<br>5′-AAACTTGAGGTCAAATGGAATGGAC-3′			
Sequencing primer	5'-GAGGGCCTATTTCCCATGATT-3'			
(g) For PCR of SSTR2-KO cell				
SSTR2-KO-PCR	F: 5′-CCTCCAGGGTCCATTAAGGT-3′<br>R: 5′-GAGGACTGCATTGCTTGTCA-3′	60	33	447
(h) For RT-qPCR validation of DEGs from pathway enrichment analysis				
OLR1	F: 5′-GAAACCCTTGCTCGGAAGCTGA-3'<br>R: 5′-CAGATCCAGTCTTGCGGACAAG-3'	60	33	131
SLC2A3	F: 5′-TGCCTTTGGCACTCTCAACCAG-3'<br>R: 5′-GCCATAGCTCTTCAGACCCAAG-3'	60	33	98
BMP2	F: 5′-TGTATCGCAGGCACTCAGGTCA-3'<br>R: 5′-CCACTCGTTTCTGGTAGTTCTTC-3'	60	35	133
CALD1	F: 5′-CTGTTCCTGCTGAAGGTGTACG-3'<br>R: 5′-CCTACCTTCAAGCCAGCAGTTTC-3'	60	33	115
CCN1	F: 5′-GGAAAAGGCAGCTCACTGAAGC-3'<br>R: 5′-GGAGATACCAGTTCCACAGGTC-3'	60	30	141
CCN2	F: 5′-CTTGCGAAGCTGACCTGGAAGA-3'<br>R: 5′-CCGTCGGTACATACTCCACAGA-3'	60	33	148
ARL4A	F: 5′-CCTGTGCAATCATAGGAGATGGC-3'<br>R: 5′-CAGAGAAAACCTACTCCACACAG-3'	60	30	146
ARID5B	F: 5′-GAATTAGGCGGTAATCCTGGGAG-3'<br>R: 5′-TCCGAGGTTTGATTGGAGGCAG-3'	60	33	133
SAMD9	F: 5′-GGGAACTACCTTGGCTATGCAC-3'	60	33	149

	R: 5′-CGTATTCCTGACGGTTCATTGCC-3'			
G0S2	F: 5′-GCCTGATGGAGACTGTGTGCAG-3'<br>R: 5′-TCCTGCTGCTTGCCTTTCTCCT-3'	60	33	142
ATF3	F: 5′-CGCTGGAATCAGTCACTGTCAG-3'<br>R: 5′-CTTGTTTCGGCACTTTGCAGCTG-3'	60	30	137
CEBPB	F: 5′-AGAAGACCGTGGACAAGCACAG-3'<br>R: 5′-CTCCAGGACCTTGTGCTGCGT-3'	60	30	125
COL12A1	F: 5′-CAGTGCCTGTAGTCAGCCTGAA-3'<br>R: 5′-GGTCTTGTTGGCTCTGTGTCCT-3'	60	35	133
VEGFA	F: 5′-TTGCCTTGCTGCTCTACCTCCA-3'<br>R: 5′-GATGGCAGTAGCTGCGCTGATA-3'	60	30	126
GADD45A	F: 5′-CTGGAGGAAGTGCTCAGCAAAG-3'<br>R: 5′-AGAGCCACATCTCTGTCGTCGT-3'	60	30	146
XAF1	F: 5′-CCTCCATGAGGCTTACTGCCTG-3'<br>R: 5′-GAAACTCCAGCGAGGACTTCTG-3'	60	35	158
BST2	F: 5′-TCTCCTGCAACAAGAGCTGACC-3'<br>R: 5′-TCTCTGCATCCAGGGAAGCCAT-3'	60	30	113
TXNIP	F: 5′-CAGCAGTGCAAACAGACTTCGG-3'<br>R: 5′-CTGAGGAAGCTCAAAGCCGAAC-3'	60	28	141

Table S2. Quality and quantity of the WGBS data

Sample ID	Total number of sequenced reads	Mapped read length (bp)*	Mapping rate (%)	Duplicate rate (%)	Genome coverage (x)	GC (%)	Q30 (%)
GM_06	870,721,304	27,008,868,396	83.2	54.60	8.61	24.15	92.2
IM _06	874,767,156	24,086,629,531	82.5	60.80	7.68	24.46	92.7
DP_06	895,996,150	43,078,415,280	77.2	24.64	13.73	24.50	91.7
GM_08	820,805,300	36,366,392,107	77.4	29.17	11.59	23.97	90.6
IM_08	908,487,140	39,955,049,794	77.9	25.70	12.74	24.39	90.2
GT_08	872,063,030	38,458,717,025	79.7	31.35	12.26	24.72	91.1
GM_28	818,544,510	40,553,800,133	80.5	29.97	12.93	24.03	91.7
IM_28	808,919,658	41,301,714,462	80.9	27.82	13.17	23.90	90.8
DP_28	799,168,270	42,091,896,856	81.5	23.88	13.42	24.40	88.9
Average	852,163,613	36,989,053,732	80.1	34.21	11.79	24.28	91.1

*The sequence reads were mapped to hg19 as the reference genome.

Table S3. Average value of selected CpGs methylation peak at promoter of *SST* and *SSTR* family genes from UCSC genome browser of WGBS data (in vertical range of 0 to 100)

Gene	Sample category	ID	Methylation peaks at CpG probes*				
			cg22277994	cg21547708	cg13344169	Mean1	Mean2
SSTR2	GM	#06	3.93	5.36	7.14	5.48	6.56
		#08	4.63	6.35	8.16	6.38	
		#28	7.13	7.13	9.23	7.83	
	IM	#06	35.19	35.96	35.96	35.70	48.94
		#08	66.11	70.85	70.85	69.27	
		#28	40.78	42.41	42.41	41.86	
	GT	#06	69.95	74.18	71.26	71.80	65.71
		#08	57.53	58.36	58.36	58.09	
		#28	69.20	74.18	58.36	67.25	

*These are CpG probes designed from 450K methylation bead chip.
□ Mean1 was calculated from three CpGs for each sample, while mean2 was calculated from mean values of three samples.
□ Two DP and one GT samples were grouped into one category, GT.

Table S4. Clinicopathologic characteristics of gastric tumors by anti-SSTR2 immunostaing using Tissue Microarray Analysis

		Negative (n=398)	Positive (n=34) [a]				*P* [b]
			+	++	+++	Sum	
Sex	Male	288	14	5	8	27	0.37
	Female	110	2	3	2	7	
Lauren [c]	Intestinal	160	9	4	7	20	0.02*
	Diffuse	168	4	1	3	8	
	Mixed	68	3	2	0	5	
	Undetermined	2	0	1	0	1	
WHO	papillary	1	1	0	0	1	0.07
	WD tub	27	1	0	1	2	
	MD	136	5	5	6	16	
	PD	144	8	1	2	11	
	mucinous	14	0	0	0	0	
	Poorly cohesive	70	0	1	1	2	
	others	6	1	1	0	2	
Depth	mucosa	46	0	0	1	1	0.05*
	submucosa	69	1	0	2	3	
	Proper muscle	63	2	1	0	3	
	subserosa	132	10	5	6	21	
	serosa	79	3	2	1	6	
	adjacent	9	0	0	0	0	
Invasion[d]	AGC	283	15	8	7	30	0.03*
	EGC	115	1	0	3	4	

[a] Staining patterns for SSTR2 were scored as 0 (negative), 1 (weakly positive), 2 (moderately positive) and 3 (strongly positive), which were grouped into negative (0) and positive (+, ++, and +++).
[b] Statistical test was analyzed by Student's *t*-test or by *Chi*-square test between Negative and Sum of Positive.
[c] The histological classification of gastric carcinoma into the intestinal type and diffuse type is based on the criteria proposed by Lauren.
[d] EGC, early gastric cancer; AGC, advanced gastric cancer

Table S5. Summary for RNA-seq in SSTR2-KO SNU638 and SSTR2-OVER AGS, MKN45, and MKN74 cells

Sample ID	Total number of sequenced reads	Total number of uniquely mapped reads[a]	RNA integrity number (RIN)	Ratio of exon-mapped reads to total uniquely mapped reads (Expression Profile Eff iciency)	Total number of detected transcripts with reads ≥1[b]
SNU638-CON-1	65,560,574	56,085,848	10	90.04%	16247
SNU638-KO-1	60,377,620	53,000,114	9.8	88.21%	16198
AGS-CON-1	75,784,864	67,734,814	10	89.38%	15909
AGS-OVER-1	50,066,306	51,879,350	10	87.88%	15749
MKN45-CON-1	49,634,116	59,071,284	10	90.10%	16255
MKN45-OVER-1	59,032,818	68,964,256	10	90.46%	16278
MKN74-CON-1	51,477,740	64,365,608	10	90.40%	16022
MKN74-OVER-1	50,417,634	65,806,472	10	90.17%	16059
SNU638-CON-2	56,699,202	46,672,294	10	89.39%	16142
SNU638-KO-2	72,979,420	44,625,714	9.8	87.82%	16101
AGS-CON-2	54,924,436	43,894,046	10	87.67%	15584
AGS-OVER-2	76,233,128	44,837,854	10	87.10%	15652
MKN45-CON-2	59,225,444	54,192,126	10	89.76%	16230
MKN45-OVER-2	50,870,376	53,518,984	10	90.36%	16154
MKN74-CON-2	71,199,990	52,655,272	10	90.55%	15914
MKN74-OVER-2	58,149,812	55,207,862	10	90.49%	16001
SNU638-CON-3	50,813,020	49,093,628	10	89.78%	16127
SNU638-KO-3	55,527,348	49,116,084	9.8	88.45%	16148
AGS-CON-3	61,006,960	43,576,902	10	87.80%	15551
AGS-OVER-3	50,517,676	44,059,878	10	87.39%	15644
MKN45-CON-3	62,288,442	49,504,062	10	90.13%	16147
MKN45-OVER-3	52,209,726	45,953,766	10	90.34%	16039
MKN74-CON-3	54,680,694	50,895,018	10	89.76%	15904
MKN74-OVER-3	60,081,296	45,756,848	10	90.58%	15863
Average	58,739,943	52,519,504		89.33%	15997

[a]As a human reference genome, hg19 was used.
[b]A higher minimum coverage threshold is permitted.

Table S6. Enrichment R analysis for DEGs in SSTR2-KO and SSTR2-OVER cells compared to control cells using MSigDB_Hallmark_2020 (Excel file)
